# Prevalence of HIV, Hepatitis B and C Virus Co-infections among Iranian High-Risk Groups: A Systematic Review and Meta-Analysis

**DOI:** 10.21315/mjms2019.26.3.3

**Published:** 2019-06-28

**Authors:** Amir Almasi Hashiani, Farzin Sadeghi, Erfan Ayubi, Shahab Rezaeian, Yousef Moradi, Kamyar Mansori, Behzad Gholamaliei, Abolfazl Mohammadbeigi, Salman Khazaei

**Affiliations:** 1 Department of Epidemiology, Arak University of Medical Sciences, Arak, Iran and Traditional and Complementary Medicine Research Center, Arak University of Medical Sciences, Arak, Iran; 2 Infectious Diseases & Tropical Medicine Research Center, Babol University of Medical Sciences, Babol, Iran; 3 Department of Community Medicine, School of Medicine, Zahedan University of Medical Sciences, Zahedan, Iran; 4 Research Center for Environmental Determinants of Health, Kermanshah University of Medical Sciences, Kermanshah, Iran; 5 Pars Advanced and Minimally Invasive Medical Manners Research Center, Pars Hospital, Iran University of Medical Sciences, Tehran, Iran; 6 Department of Epidemiology and Biostatistics, School of Medicine, Zanjan University of Medical Sciences, Zanjan, Iran; 7 Department of Public Health, School of Public Health, Hamadan University of Medical Sciences, Hamadan, Iran; 8 Research Center of Gastroenterology and Hepatology, Qom University of Medical Sciences, Qom, Iran; 9 Research Center for Health Sciences, Hamadan University of Medical Sciences, Hamadan, Iran

**Keywords:** HIV infection, hepatitis B, hepatitis C, co-infection

## Abstract

Co-infection with hepatitis B and C among HIV infected patients are prevalent among high-risk populations. This meta-analysis aimed to estimate the prevalence of HIV, HCV and HBV co-infections among high-risk populations in Iran.

We systematically searched the national and international electronic databases until 2016. The primary outcome was the prevalence of HIV, HBV, HCV and HIV co-infections in different high-risk populations in Iran. All English and Persian studies conducted on Iranian high-risk groups were included in the study. The review was reported based on PRISMA guidelines and data were analysed at 95% confidence level using random effect models.

Overall, 916 relevant papers were recognised and 14 articles were included in the meta-analysis. The pooled estimates of HBV/HCV, HCV/HIV, HBV/HIV and HBV/HCV/HIV were 1.3% (95%CI: 0.5–2.1), 16.3% (95%CI: 1.1–31.6), 0.5% (95%CI: 0–1.4) and 0.5% (95%CI: 0.2–0.8), respectively. Based on subgroup analysis, there was a higher proportion of all co-infections from the years 2010–2016 as compared to that of the years 2003–2009.

Our results highlighted that HCV/HIV co-infection in Iranian high-risk groups including injection drug users (IDUs) and prisoners is common. In addition, the increasing trend of coinfections should be considered alarming for policymakers.

## Introduction

The prevalence and burden of HIV infection varies widely among developed and developing countries (1). This proportion also varies across the different regions in each country. HIV infection is a major public health problem and is also a major cause of morbidity and mortality in developing countries (2). Accordingly, it is suggested that a high-impact prevention approach should be conducted in communities with a high proportion of HIV infection (3).

Co-infection with hepatitis B and C among HIV infection patients are more prevalent (4) especially among high-risk groups such as prisoners and people who inject drugs (PWID) (5, 6). In addition, these infections have been reported as one of the most important causes of liver-related morbidity and mortality worldwide (7, 8).

It has been recently shown that there is a relatively low prevalence of HIV infection and other blood-borne viral infections in the Iranian general population. This low prevalence is attributable to widespread prevention strategies across the country e.g. harm reduction services, methadone maintenance therapy, public awareness on routes of infection transmission, and free HIV testing and counseling services. On the other hand, despite prevention strategies such as HCV screening in HIV-infected patients and high-risk groups, Iran is faced with an HIV epidemic among high-risk populations e.g. PWID, with a prevalence of 18.4% (9).

Several studies were conducted in Iran in order to estimate the prevalence of HIV, HBV and HCV co-infections among prisoners and other high-risk groups; however, the results are inconsistent and the actual prevalence rate among high-risk population is unclear. Thus, the aim of this meta-analysis was to estimate the prevalence rate of HIV, HCV and HBV coinfections among high risk populations in Iran.

## Material and Methods

### Search Strategy

Two investigators searched the following international electronic bibliographic databases including: PubMed, Scopus and ISI; and national databases including: Magiran, Iranmedex and SID from year 2000 to end of year 2016. A manual search of the reference lists of published articles was also performed. This review was performed in accordance with the PRISMA (Preferred Reporting Items for Systematic Reviews and Meta-Analyses) statement issued in 2009 (10).

Key search terms included terminology for prostitute; FSW or sex worker; intravenous drug users; drug addicts; IDU or injection drug users; prisoner; jail; inmate or prison; HIV or human immunodeficiency virus, HBV or hepatitis B, HCV or Hepatitis C or blood borne infection; and Iran.

All English and Persian papers that were conducted on Iranian high-risk groups to determine the prevalence of HIV, HCV and HBV infection were included in the study. Papers with no relevant data, duplicate papers, review articles, randomised clinical trials and no high-risk group studies were excluded from the meta-analysis.

We defined sex worker as exchanging sex for money, drugs, or goods (11). Also IDUs were defined as people who inject narcotic substances into the body with a hollow needle and a syringe which is pierced through the skin into the body, usually intravenously (12). We included English or Farsi cross-sectional articles on Iranian high-risk groups that determined HBV, HCV and HIV infection in patients based on the laboratory criteria according to national guideline definitions. Therefore, measured HIV (HIV Ab) and co-infections such as HBV (HBs Ag) or HCV (HCV Ab) infection with confirmatory lab tests were included. The articles with no relevant data or other design were excluded.

### Data Extraction and Quality Assessment

Two independent authors (AAH and SK) reviewed the retrieved studies and the following information were extracted: (i) name of the first author, (ii) publication year and location of study, (iii) total sample size, (iv) the reported prevalence of co-infection, (v) recruitment setting, (vi) recruitment method, (vii) age group and (viii) high risk group (IDUs, FSWs, or prisoners). Duplications were identified by comparing detailed study characteristics, including author names, study period, study location, the number of infection cases, and the sample size. If two publications were found to be from the same data source, only the earlier publication was included.

The Kappa agreement (95%) was used to identify the inter-authors reliability. The value of Kappa over 0.75 indicates excellent inter-authors reliability or inter-authors agreement. The third author (AAH) was considered as arbiter to resolve any disagreements. The quality of the studies was evaluated by two independent reviewers (AAH and SK) according to the Newcastle–Ottawa scale (13).

### Statistical Analysis

Meta-analyses were carried out using Stata software, version 12 (Stata Corp, College Station, TX, USA). We did meta-analysis for each HIV, HCV, HBV, HBV/HCV, HBV/HIV, HCV/ HIV and HBV/HCV/HIV prevalence in every subpopulation. The variance of each study was calculated through the variance of the binomial distribution given that the prevalence rate had a binomial distribution. Then, each study was given a weight, which was inversely proportional to the variance.

The analysis of heterogeneity was performed using the Cochran’s Q test (with *P*-value < 0.10) and it was quantified by the I^2^ statistic. An I^2^ value of 50 % or greater and/ or a Q-statistic value of *P* < 0.05 suggest the presence of heterogeneity, which means that differences in the point estimates reported by the included studies are greater than one would expect due to chance alone. Thus, pooling of these data using a fixed-effects meta-analysis would be invalid. Tau-squared (t^2^ or Tau^2^) statistic and Egger’s linear regression test were used to explore the between-study variance and to investigate publication bias, respectively. Subgroups analysis was conducted on the basis of infection type, high-risk group and geographical regions, and to examine the impact of moderator variables on prevalence rate meta-regression was used.

In order to calculate a weighted mean estimate of prevalence for mentioned infections across included studies, prevalence estimates by each study were pooled using a random-effects meta-analysis model at a confidence level of 95%.

## Results

**Figure 1:**
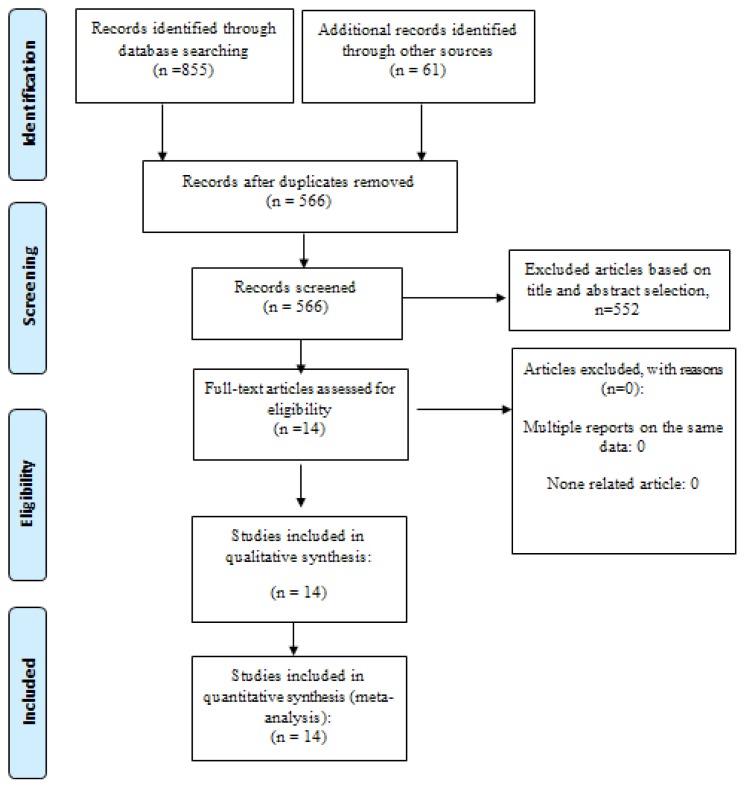
Flow diagram of study selection

Figure 1 depicted the process of the search and study selection (10). In total, 916 relevant papers were recognised from the searches in different national and international databases. After excluding duplicates, 566 papers remained and then we disqualified 552 papers by screening titles and abstracts. Finally, 14 articles were included in the meta-analysis (14–27).

### Study Characteristics

The characteristics of primary studies that were included in this meta-analysis are shown in Tables 1 and 2. These papers were published between 2003 and 2016 with various sample sizes which ranged from 70 to 1444, with a total of 6218 cases with high-risk behaviour including IDUs (six studies), prisoners (six studies), HIV positive (one study), and drug addicts (one study).

## Evaluation of Heterogeneity and Meta-Analysis

**Figure 2:**
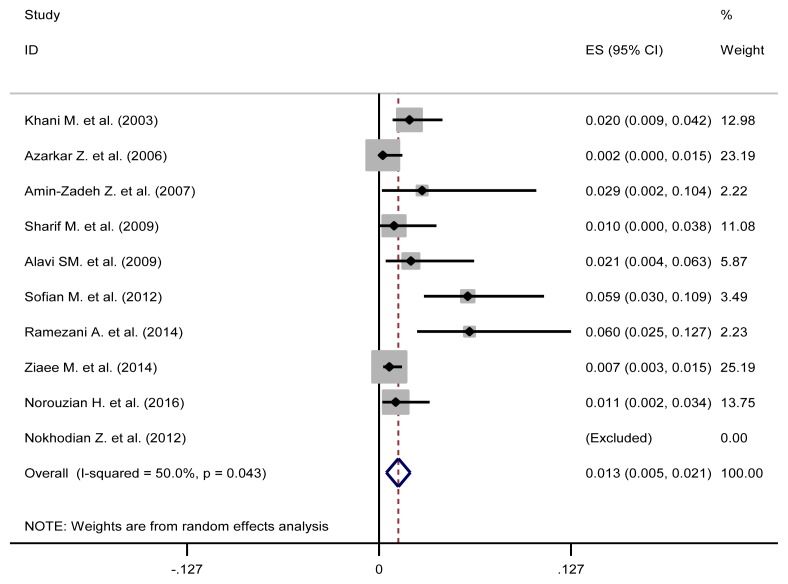
Forest plot showing prevalence of HBV/HCV comorbidities in the Iranian high-risk group

**Figure 3:**
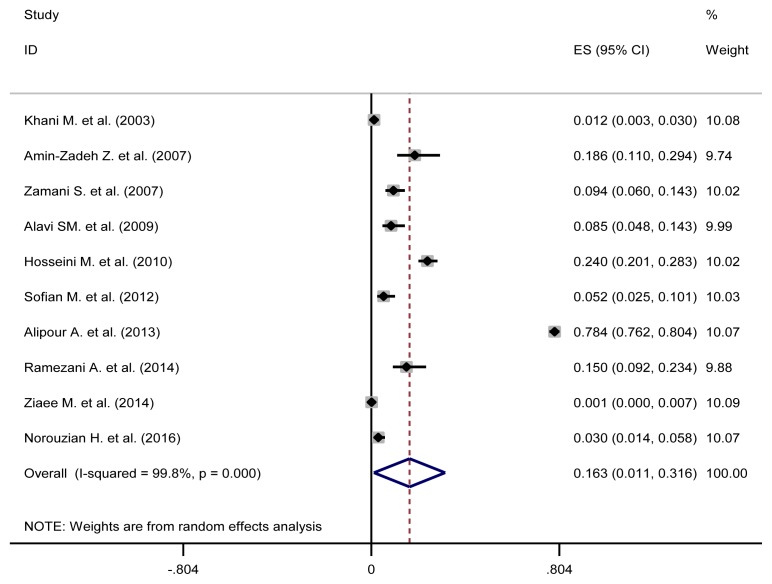
Forest plot showing prevalence of HCV/HIV comorbidities in the Iranian high risk group

**Figure 4:**
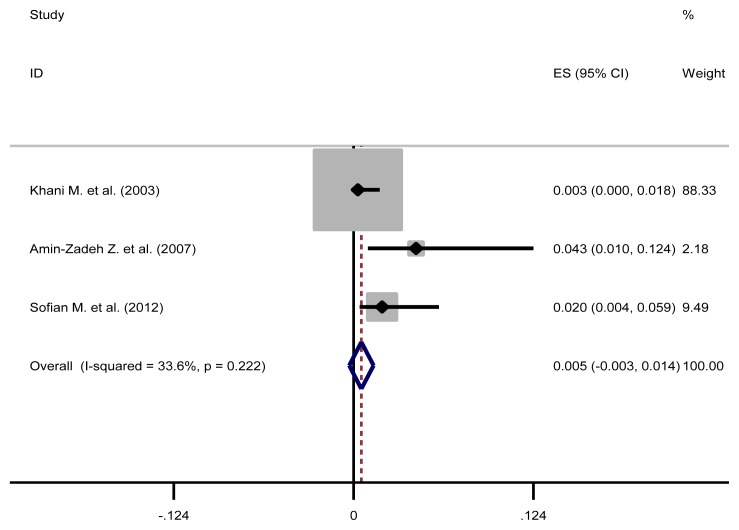
Forest plot showing prevalence of HBV/HIV comorbidities in the Iranian high risk group

**Figure 5:**
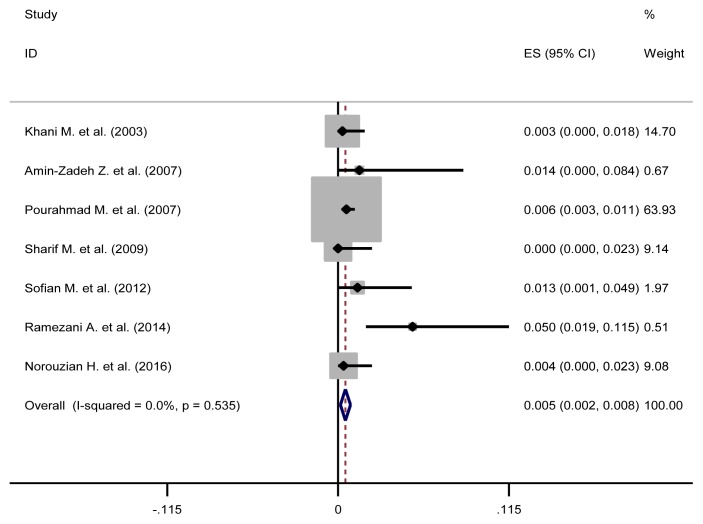
Forest plot showing prevalence of HBV/HCV/HIV comorbidities in the Iranian high risk group

The pooled estimate of HBV/HCV, HCV/ HIV, HBV/HIV and HBV/HCV/HIV were 1.3% (95%CI: 0.5–2.1), 16.3% (95%CI: 1.1–31.6), 0.5% (95%CI: 0–1.4) and 0.5% (95%CI: 0.2–0.8), respectively (Table 3 and Figures 2, 3, 4 and 5). The results of Cochran’s Q test and I^2^ statistics suggested a significant heterogeneity among the included studies. Considering significant heterogeneity between studies, we used a random effect model and we also performed a subgroup analysis based on time period (Table 4).

## Publication Bias

On the basis of the Begg’s test, there were some evidence of publication bias in HBV/HCV co-infection (*P* = 0.012), whereas there was no publication bias in HCV/HIV (*P* = 0.176), HBV/ HIV (*P* = 0.117) and HBV/HCV/HIV (*P* = 0.091) co-infections. Also, the results of funnel plot showed that there was some asymmetry in the point estimate of published papers.

## Sensitivity Analysis

The prevalence of HCV/HIV co-infection in the Alipour et al. (27) study was very high (78%). When we excluded their study, the pooled prevalence of HCV/HIV co-infection decreased to 8.33% (95%CI: 5.02–11.64) from 16.3% (95%CI: 1.1–31.6), and in the studies between 2010 and 2016 it was decreased to 9.05% (95%CI: 2.54–15.5) while before excluding Alipour et al., it was 21.0% (95%CI: 0–49.1).

## Subgroup Analysis

Based on the results, there was considerable heterogeneity between studies and therefore subgroup analysis based on time period (2003– 2009 and 2010–2016) was done for all coinfections and their results were displayed in Table 4. The results showed that the pooled prevalence of all co-infections in the second period (2010–2016) were higher compared to the first period.

## Discussion

Despite decades of countrywide preventive strategies aimed to reduce the burden of main blood-borne infections, HBV and HCV are still two major causes of morbidity and mortality in HIV-infected individuals in Iran. Most characteristically, the natural history of HBV and HCV related liver disease and progression to hepatocellular carcinoma (HCC) is accelerated due to HIV co-infection (28). Determining the prevalence of HCV/HIV, HBV/HIV and triple coinfections in Iranian high-risk groups could be helpful to develop more effective interventions against aforementioned blood-borne viruses. The results of our study demonstrated that in Iranian high-risk groups including IDUs and prisoners, one in six HIV positive patients were concurrently suffering from HCV infection. According to recently published global meta-analysis, the prevalence of HCV/HIV co-infection is highest among people who inject drugs (PWID) as compared with other high-risk groups and the general population (29). In global estimates of HCV/HIV co-infection, the greatest burden belongs to Eastern Europe and Central Asia due to the large HIV-infected population of PWID (29). Our findings confirm previously published evidence from Iran on the seriousness of HCV/HIV co-infection in PWID and prisoners. Bagheri Amiri et al., in their recent systematic review study, reported that HCV/HIV co-infection prevalence was 10.95% in Iranian PWID and 1.71% in Iranian prisoners (30). Our results are close to what they reported if we estimate the prevalence in each high-risk group separately after excluding the Alipour et al. study (31). The prevalence of HCV/HIV co-infection in the Alipour et al. study was very high (78%). When we excluded the aforementioned study after sensitivity analysis, the pooled prevalence of HCV/HIV co-infection decreased considerably. Currently, health policy makers in Iran have given so much attention to HIV, while failing to provide proper care to curable comorbidities for example HCV.

In the present study, the pooled prevalence of HBV/HIV co-infection in Iranian high risk groups was 0.5%. Matthews et al., in their recent review study, reported that prevalence of HIV-HBV co-infection were 1.1%–35.7% in sub-Saharan African cohorts (28). In addition, Agyeman et al., in their recently published meta-analysis reported 13.6 % prevalence rate for HIV-HBV co-infection in Ghana (32). HBV/HIV co-infection is regarded as a particular challenge in Sub-Saharan Africa due to high prevalence of HIV and high HBV endemicity. Our results are inconsistent with a recently published study from Iran, which reported 1.8 % and 0.13% prevalence rate for HBV-HIV co-infection among injecting drug users and prisoners, respectively. It should be noted that subgroup analysis based on time period showed that our results was close to the findings of the aforementioned study (30).

Triple infection with HIV, HBV and HCV has clinically more unfavorable consequences than mono- or dual infections (33). This scenario is uncommon globally and in sub-Saharan Africa (28). In most Sub Saharan populations triple infection with HIV, HBV and HCV was reported as ≤ 1% (34–36). In the current study, pooled prevalence of triple infection with HIV, HBV and HCV in Iranian high risk groups was 0.5%. Our results are in agreement with global reports and findings from Iranian high risk populations (28, 30).

A number of limitations exist in the current study that should be noted. First, there was a considerable heterogeneity between studies which we tried to resolve by subgroup analysis. Second, different types of kits were utilised for detecting HIV Ab, HCV Ab and HBs Ag which may affect the obtained results of the current systematic review. Third, occult HBV infection, which is characterised by a negative HBs Ag with detectable HBV DNA in the serum, may result in a false-negative HBs Ag test, making the HBV results more difficult to interpret.

## Conclusion

Our results highlighted the seriousness of HCV/HIV co-infection in Iranian high-risk groups including IDUs and prisoners. The significance of HCV among HIV-positive patients must receive greater attention and strategic policy directions including healthcare and clinical care delivery can help to reduce resultant morbidity and mortality among Iranian high-risk groups. In addition, the increasing trend of coinfections should be alarming for policymakers. Accordingly, screenings of co-infections as an important strategy among high-risk groups will not only prevent new infection-related morbidity, but will also reduce further transmission in the community.

## Figures and Tables

**Table 1:** Description of the studies included in the meta-analysis

First author	Year	Location	High risk group	Recruitment setting	Recruitment method	Age group	Sex
Khani M et al.	2003	Zanjan	Prisoners	Prison	Unknown	33.7 ± 10.2	Male
Azarkar Z et al.	2006	Birjand	Prisoner	Prison	Stratified random sampling	34.1 ± 11.7	Both
Zamani S et al.	2007	Tehran	IDU	DIC and neighboring parks and streets	Unknown	median 32	Both
Pourahmad M et al.	2007	Esfahan, Chaharmahal and Lorestan	Prisoner	Prison	Unknown	25–60	Male
Aminzadeh Z et al.	2007	Tehran	IDU	Hospital	Volunteers	34.4 ± 9.6	Male
Alavi SM et al.	2009	Ahvaz	IDU	Hospitalized	Unknown	26.3 ± 5.7	Both
Sharif M et al.	2009	Kashan	IDU	Hospitalized	Volunteers	36.5 ± 10.2	Both
Hosseini M et al.	2010	Tehran	IDU	temporary detention center	Census	NA	Male
Sofian M et al.	2012	Arak	Prisoner	Prison	Census	30.7 ± 5.9	Male
Nokhodian Z et al.	2012	Isfahan	Prisoner	Prison	Census	34.54 ± 11.2	Female
Alipour A et al.	2013	Shiraz	HIV Possitive	Counselling Centre for Behav Dis	Census	38.4 ± 9.2	Both
Ziaee M et al.	2014	Southern Khorasan	Prisoner	Prison	random sampling	34.7 ± 11.4	Both
Ramezani A et al.	2014	Arak	IDU	MMT center	Census	median 33.3 (R: 17–58)	Male
Norouzian H et al.	2016	Lorestan	Addicts	Drug Treatment Centers	Volunteers	31.7	Both

**Table 2:** Description of the studies included in the meta-analysis

First author	Sample size	HCV/HIV (N)	HCV/HIV (%)	HBV/HIV (N)	HBV/HIV (%)	HBV/HCV (N)	HBV/HCV (%)	HBV/HCV/HIV (N)	HBV/HCV/HIV (%)
Khani M et al.	346	4	1.16	1	0.28	7	2.02	1	0.29
Azarkar Z et al.	400	-	-	-	-	1	0.25	-	-
Zamani S et al.	202	19	9.41	-	-	-	-	-	-
Pourahmad M et al.	1431	-	-	-	-	-	-	8	0.56
Aminzadeh Z et al.	70	13	18.57	3	4.28	2	2.86	1	1.43
Alavi SM et al.	142	12	8.45	-	-	3	2.11	-	-
Sharif M et al.	200	-	-	-	-	2	1.00	0	0
Hosseini M et al.	417	100	23.98	-	-	-	-	-	-
Sofian M et al.	153	8	5.23	3	1.96	9	5.88	2	1.31
Nokhodian Z et al.	161	-	-	-	-	0	0	-	-
Alipour A et al.	1,444	1,132	78.39	-	-	-	-	-	-
Ziaee M et al.	881	1	0.11	-	-	6	0.68	-	-
Ramezani A et al.	100	15	15.00	-	-	6	6.00	5	5.00
Norouzian H et al.	271	8	2.95	-	-	3	1.11	1	0.37

**Table 3:** Summary of meta-analysis results

Comorbidities	Meta-analysis			Heterogeneity		
	Prevalence	95%CI	Model	Statistic	*P*-value	I^2^
HBV/HCV	1.3%	0.5–2.1	Random	15.99	0.043	50.0%
HCV/HIV	16.3%	1.1–31.6	Random	5237.71	0.001	99.8%
HBV/HIV	0.5%	0–1.4	Fixed	3.01	0.222	33.6%
HBV/HCV/HIV	0.5%	0.2–0.8	Fixed	5.07	0.535	0.0%

**Table 4:** Subgroup meta-analysis based on time periods

Type of co-morbidity	Time period	No. of studies	Meta-analysis			Heterogeneity		
			Pooled estimate	95%CI	*P*	Statistic	I^2^	*P*
HBV/HCV	2003–2009	5	1.0%	0.1–1.9	0.031	5.43	26.3%	0.246
	2010–2016	4	2.2%	0.3–4.1	0.026	10.39	71.1%	0.016
HCV/HIV	2003–2009	4	8.5%	1.9–15.0	0.011	32.27	90.7%	0.001
	2010–2016	6	21.0%	0–49.1	0.144	5205	99.9%	0.001
HBV/HIV	2003–2009	2	1.3%	0–4.6	0.462	1.85	46.0%	0.174
	2010–2016	1	2.0%	0–4.7	0.159	0.00	0%	-
HBV/HCV / HIV	2003–2009	4	0.5%	0.1–0.8	0.013	1.18	0%	0.757
	2010–2016	3	1.2%	0–3.1	0.190	3.64	45.1%	0.162
